# Synergistic effect of wire bending and salivary pH on surface properties and mechanical properties of orthodontic stainless steel archwires

**DOI:** 10.1186/s40510-015-0109-6

**Published:** 2015-10-26

**Authors:** Marieke G. Hobbelink, Yan He, Jia Xu, Huixu Xie, Richard Stoll, Qingsong Ye

**Affiliations:** Orthodontic Department, College of Medicine and Dentistry, James Cook University, Cairns, Australia; School of Dentistry, University Medical Centre Groningen, Groningen, The Netherlands; School and Hospital of Stomatology, Wenzhou Medical University, Wenzhou, China

**Keywords:** Stainless steel archwire, Surface roughness, Young’s modulus, Maximum force, Salivary pH, Corrosion

## Abstract

**Background:**

The aim of this study was to investigate the corrosive behaviour of stainless steel archwires in a more clinically relevant way by bending and exposing to various pH.

**Methods:**

One hundred and twenty pieces of rectangular stainless steel wires (0.43 × 0.64 mm) were randomly assigned into four groups. In each group, there were 15 pieces of bent wires and 15 straight ones. Prior to measurements of the wires, as individual experimental groups (group 1, 2, and 3), the wires were exposed to artificial saliva for 4 weeks at pH 5.6, 6.6, and 7.6, respectively. A control group of wires (group 4) remained in air for the same period of time before sent for measurements. Surface roughness (Ra-value) was measured by a profilometer. Young’s modulus and maximum force were determined by a four-point flexural test apparatus. Scanning electron microscopy was used to observe the surface morphology of straight wire. Differences between groups were examined using a two-way analysis of variance (ANOVA).

**Results:**

Mean surface roughness values, flexural Young’s moduli, and maximum force values of bent wires are significantly different from those of the straight wires, which was the main effect of wire bending, ignoring the influence of pH. A significant effect was found between Ra-values regarding the main effect of pH, ignoring the influence of shape. There was a significant interaction effect of bending and pH on flexural Young’s moduli of stainless steel archwires, while pH did not show much impact on the maximum force values of those stainless steel wires. Bigger surface irregularities were seen on SEM images of straight wires immersed in artificial saliva at pH 5.6 compared to artificial saliva at other pH values. Surface depth (Rz) was more sensitive than Ra in revealing surface roughness, both measured from 3D reconstructed SEM images. Ra showed a comparable result of surface roughness to Ra-value measured by the profilometer.

**Conclusions:**

Bending has a significant influence on surface roughness and mechanical properties of rectangular SS archwires. pH plays a synergistic effect on the change of mechanical properties of stainless steel (SS) wires along with wire bending.

## Background

Stainless steel (SS) has been introduced around 1930 as an alloy for the fabrication of orthodontic archwires and is still a broadly used appliance in orthodontic practice [1]. Stainless steel is a convenient material to act as a spring because it is, among other materials, a ductile material and it is able to maintain its shape after bending. This behaviour of a SS alloy can be explained by consideration of its mechanical properties. Examination of mechanical properties is the key to understand the clinical application of a material since mechanical properties determine the behaviour of the wire in generating forces and thus influence tooth movement.

**Fig. 1:**
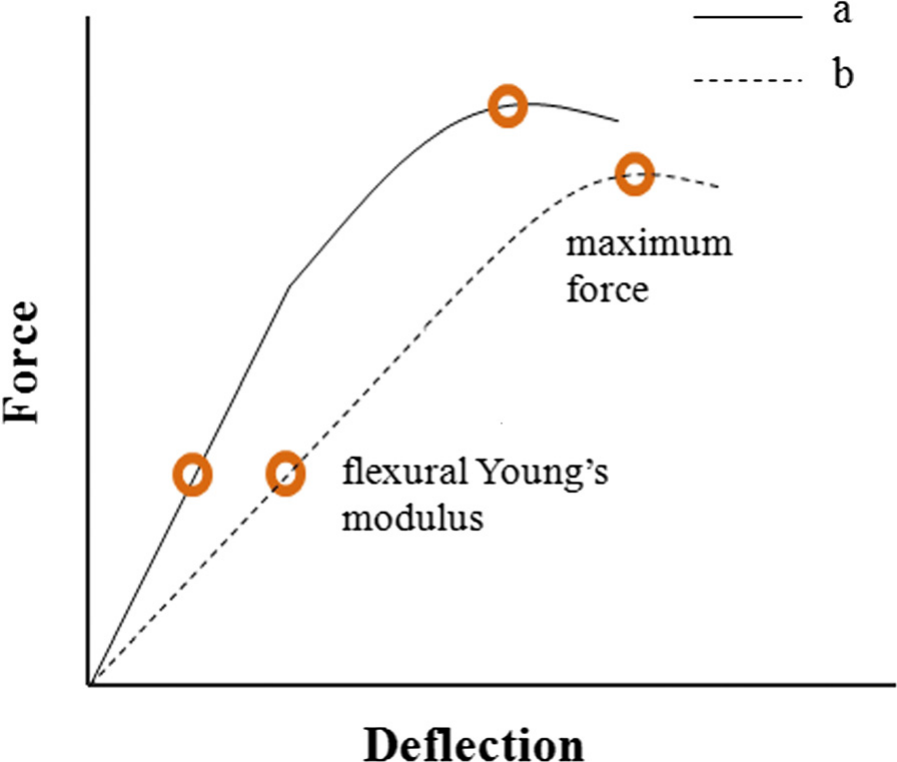
Typical force-deflection curves of an elastic material. The flexural Young’s modulus and maximum force value are indicated as *brown circles*. The flexural Young’s modulus is calculated as the slope of the linear portion of the curve by taking into account the dimensions of the wire. The maximum force is the maximum amount of force a material can withstand before it starts to fail. Curve *a* represents a stiffer material than curve *b*

An important parameter is the flexural Young’s modulus (Fig. 1). It represents the flexibility of an archwire and can be calculated as the slope of a force-deflection curve. Researchers have been reporting a flexural Young’s modulus of ~120–217 GPa [2] for as-received stainless steel archwires. Another important parameter is the maximum force. This is defined as the maximum amount of force the wire can withstand before it starts to fail [3].

Mechanical properties of stainless steel orthodontic archwires can be influenced by a variety of causes, for example, corrosion [4, 5]. Electrolytic corrosion of orthodontic appliances in the mouth regularly occurs due to the wet oral environment [6]. The pH value of saliva in the oral environment has a significant effect on the corrosion rate [7]. The resting pH of human saliva ranges from 5.6 to 7.6, which can be caused by dietary habits [8] and internal stimuli like vomitingor by diseases [9, 10].

Corrosive damage to archwires can result in deterioration of its mechanical properties. Limited studies have been performed to investigate the influence of corrosion on mechanical properties of stainless steel archwires [4, 5, 11]. The corrosion phenomenon may not only influence the mechanical properties of the orthodontic archwires but also affect surface properties, such as the surface roughness and surface texture [7, 12].

A variety of experiments have measured the surface roughness of as-received stainless steel archwires [10], resulting in a description of the surface of SS archwires as smooth, compared to titanium archwires [13, 14]. Regarding corrosion resistance, literature describes that SS archwires corroded more than titanium archwires [7, 15]. Surface roughness and texture increase with the increase of incubation time in acid solutions [13] and the decrease of pH [7].

Surface texture can be observed by a scanning electron microscope (SEM). SEM images of as-received SS archwires showed an inhomogeneous surface with different surface irregularities [2, 16], scratches, and pits [17]. SEM images of in vitro corroded SS archwires show pitting corrosion, localized corrosion, and scratches [13]. An increase in variety, type, and number of surface irregularities were observed due to wear and friction of archwires from in vivo studies [16, 18].

The corrosion phenomenon influences the mechanical properties and surface properties of stainless steel orthodontic archwires [19]. Such degradation processes can have serious clinical implications resulting in weakening the efficacy of the force delivering system. It also increases the potential for failure [15]. Previous experimental set-ups had been performed to investigate the influence of corrosion on the mechanical properties and surface properties of SS archwires [13]. However, those studies investigated straight archwire pieces only. In orthodontic clinic, archwire bending is an integral part of orthodontic treatment. Straight archwires are hardly used in a patient’s mouth. Bending causes stress in the material, which alters the wire properties and its behaviour upon challenges, e.g., corrosion.

So, the aim of this study was to investigate the corrosive behaviour of bent SS archwires in a more clinically relevant manner comparing the bent with the as-received straight wires in a clinically relevant pH solution.

## Methods

### Wire preparation

Rectangular stainless steel orthodontic archwires, 0.43 × 0.64 mm (3M Unitek, USA), were cut into pieces with a distal-end cutter (Hu-Friedy, USA) for further sample preparation. A curved shape was bent following the Ovoid Arch Form OrthoForm™ III (3M Unitek, USA) with an Adams Plier (678-320-U5 Hu-Friedy, USA). The length of the pieces was measured with an electronic digital calliper to ±0.1 mm accuracy. The total sample size of 120 pieces, 60 pieces per wire shape type, was determined by a multiple comparisons Power Analysis (Tukey-Kramer) test. These archwires were randomly divided into four groups, three experimental groups and one control group. Each group contained 30 pieces of wires, including 15 bent wires and 15 straight wires (24 mm). Additionally, one short straight wire (10 mm) was included for each group to study surface characteristics under a scanning electron microscope.

### Immersing experiment

A total of 1 L of artificial saliva was prepared (0.400 g/L NaCl, 0.400 g/L KCl, 0.795 g/L CaCl_2_, 0.010 g/L Na_2_S9H_2_O, 1.000 g/L CH_4_N_2_O, 0.789 g/L KH_2_PO_4_) and divided into three portions equally, which were calibrated to pH 5.6, 6.6, and 7.6, respectively. Experimental groups were marked as groups 1, 2, and 3 in the same order as pH values. The wires were immersed in 20 mL artificial saliva and placed in the incubator (SANYO Electric Biomedical Co., Japan) at 37 °C for 4 weeks. The solution was refreshed every week to ensure a constant pH. The wires of group 4 were exposed to air at room temperature, served as the control group.

### Surface roughness test

**Fig. 2:**
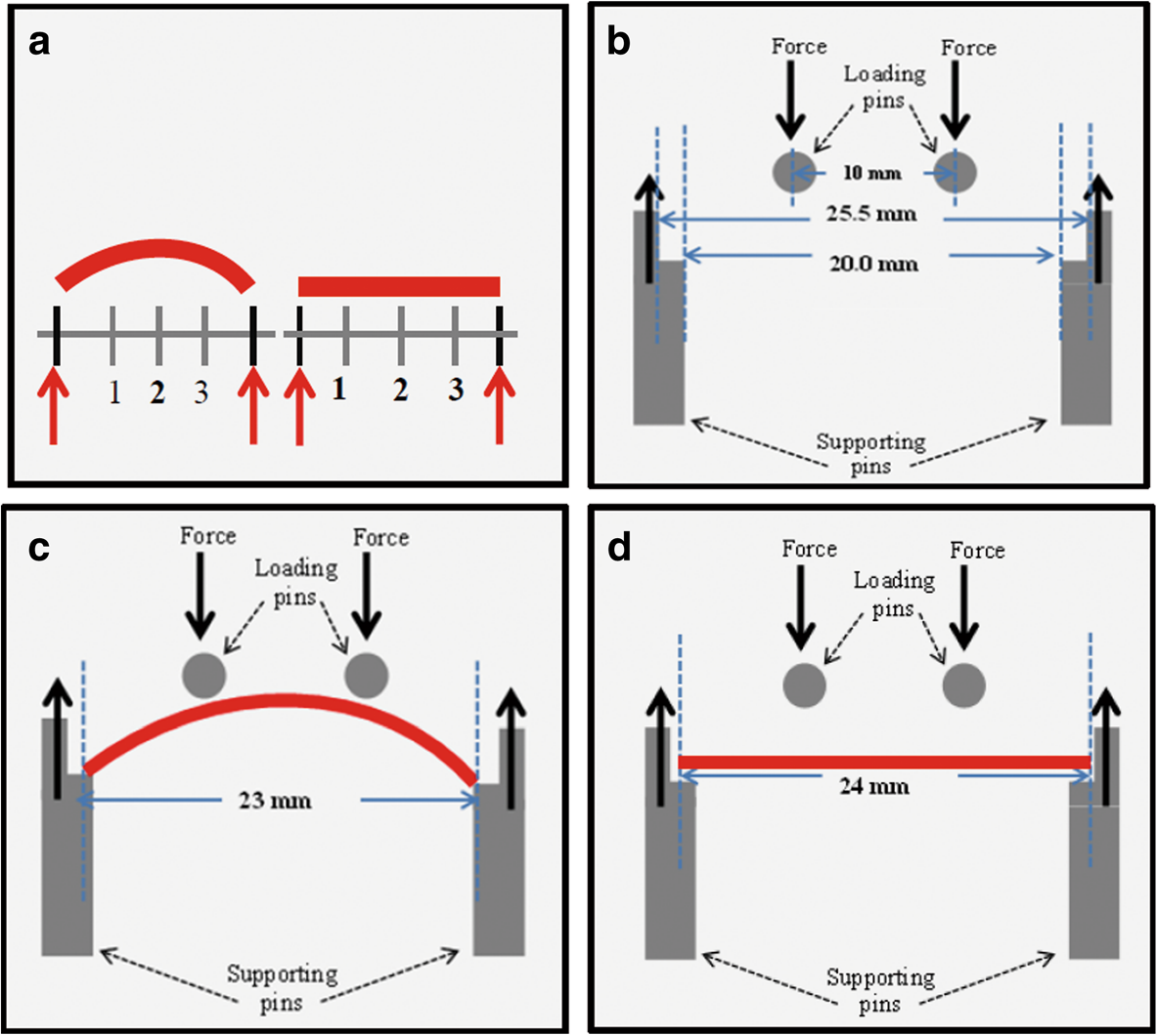
Schematics of measurements of surface roughness and mechanical properties. **a** Schematic of surface roughness of bent and straight wires measurement by a profilometer. Wire placed in-between two outer lines, indicated by *red arrows*. Bent wires were measured at around position *2* and straight wires were measured at positions *1*, *2*, and *3*. **b** Schematic drawing of the four-point flexural test apparatus for measurements of flexural Young’s modulus and maximum force of bent and straight wires. The distance between the supporting pins ranges from 20 to 25.5 mm (support span), and the distance between the loading pins is 10 mm (load span). Both loading pins move downward to produce force on the wires. **c–d** Bent and straight wire placed in-between the supporting pins, where the 0.43-mm side of wires are resting upon

Surface roughness test was carried out by a calibrated contact profilometer (RTD-200 Portable Surface Roughness tester, New Star, Australia). Mean surface roughness values (Ra-values, μm) are defined as the arithmetic mean of the absolute departures of the roughness profile from the mean line. The software of profilometer was set at a Gauss roughness filter type with a cut-off length of 0.25 mm and an assessment length of 0.75 mm. The measurement was performed by a diamond point (*φ* = 5 μm) moving along the wire with a traversing speed of 0.135 mm/s at a pressing force of 4 mN. Prior to surface roughness test, the wires were taken out of the immersion solution and air-dried on a paper towel. Each wire was measured at three different positions. To ensure the reliability of measurements, these positions were indicated by lines as shown in Fig. 2a. The mean of three Ra-values per wire was used for statistical analysis.

### Four-point flexural test

**Fig. 3:**
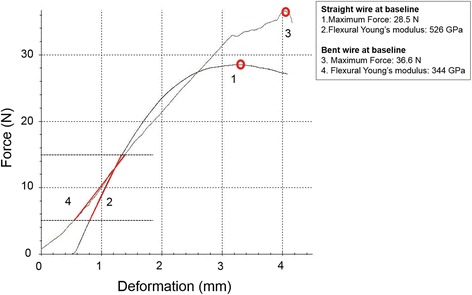
Force-deflection curves of two SS wires obtained by four-point flexural testing. The flexural Young’s moduli were the slope of the section on the curve indicated by a *red line* and the maximum force values are *circled* in *red*. Their corresponding values are given in the legend

In this research, the flexural test was performed by a Z2.5 ZwickiLine Universal Testing Machine (Zwick/Roell, Germany) with a customized four-point appliance (Fig. 2b), which consists of two loading pins and two supporting pins. A 500-N maximal force, 2-mV/V sensitivity load cell (XforceP, Zwick/Roell, Germany), containing two loading pins, was installed in the machine with a crosshead speed of 1 mm/min. The load span, distance between both loading pins, was set at 10 mm. Loading pins deformed the wire at a speed of 1 mm/min until a threshold of 25 N was reached (Fig. 2). The load-deflection data obtained from the test were plotted as force-deflection curves, and the flexural Young’s modulus was calculated by the computer software testXpert II (Zwick/Roell, Germany) (Fig. 3).

### Scanning electron microscopy

The surface of 10-mm straight wires was observed by SEM apparatus (G2 Pro, Phenom-World BV, The Netherlands) on its 0.43-mm side with a ×2000 magnification. Three places on each sample were randomly chosen for image scanning. The SEM software (Phenom Pro Suite, Phenom-World BV, The Netherlands) was used to quantity the surface morphology by reconstructing a 3D surface, from which mean surface roughness values (Ra) and mean surface depth values (Rz) were calculated. Three different regions of each image were selected for 3D reconstruction and calculation. Ra is the arithmetic mean of the absolute departures of the roughness profile from the mean line. Rz is the roughness depth, calculated as the sum of the height of the highest profile peak from the mean line and the depth of the deepest profile valley from the mean line.

### Statistical analyses

All data were analysed by SPSS 22 (IBM^®^ SPSS^®^, USA). A two-way analysis of variance (ANOVA) test was carried out for surface roughness (Ra-values), flexural Young’s moduli, and maximum force values, followed by post hoc multiple comparison tests. A significance level of *p* = 0.05 was used.

## Results

### Surface roughness

**Fig. 4:**
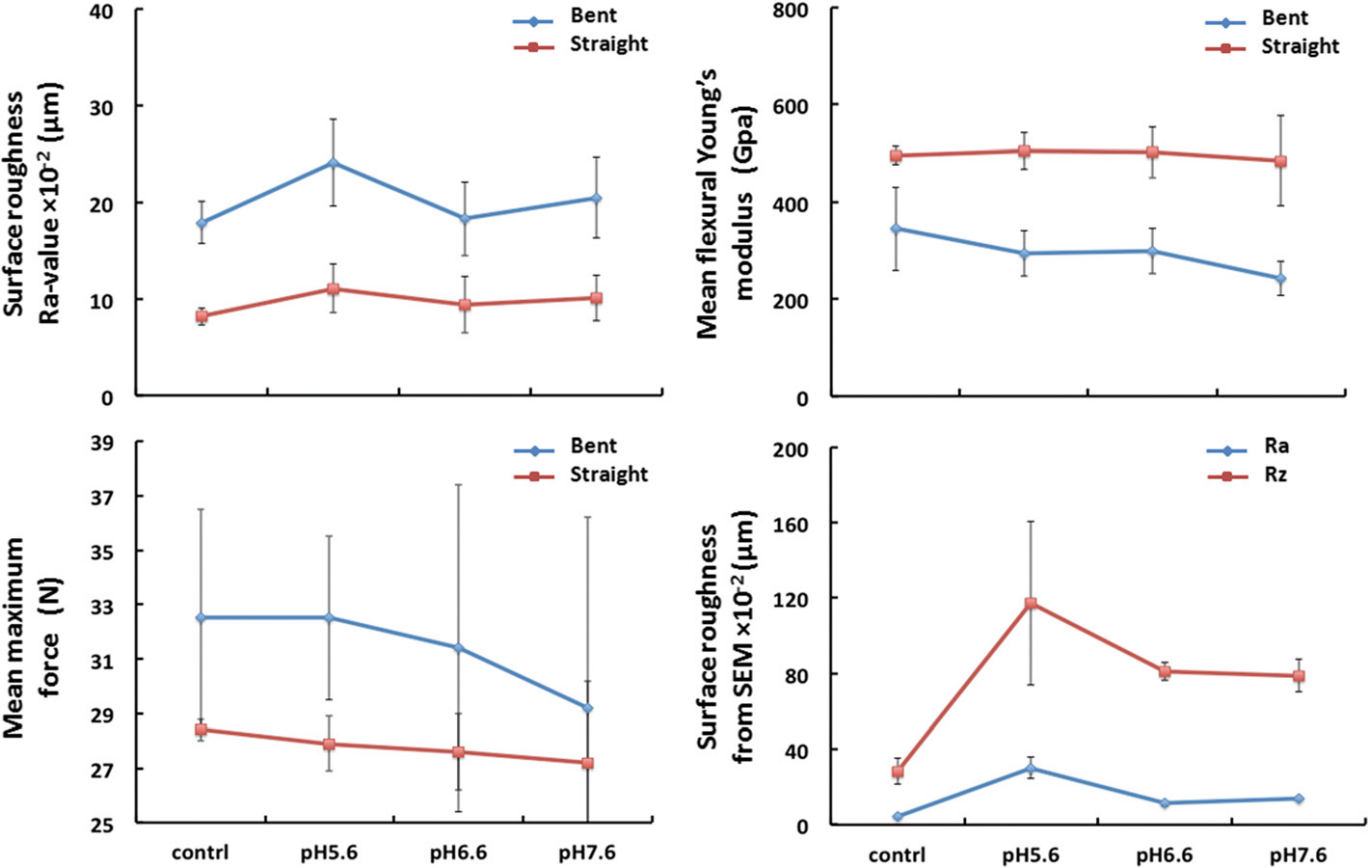
Comparison of surface roughness, Young’s modulus, and maximum force of bent and straight wires. Surface roughness (Ra-value, μm) of bent and straight SS archwires was measured by a profilometer; flexural Young’s modulus (GPa) and maximum force (N) of bent and straight SS archwires were measured by a four-point bending test; surface roughness (Ra and Rz,μm) of straight SS wires were measured via 3D reconstruction of SEM images. All measured wires were exposed to air or immersed in artificial saliva at pH 5.6, 6.6, and 7.6 for 4 weeks according to protocol

The Ra-value of the bent wires was significantly higher than that of the straight wires in all three pH groups (*p* < 0.000), where the straight wires in the control group had the lowest Ra-value (8.2 ± 0.9) × 10^−2^ μm and the bent wire had the roughest surface (24.1 ± 4.5) × 10^−2^ μm after being immersed in artificial saliva of pH 5.6 for 4 weeks (Table 1). Surface roughness from different pH groups showed highly varied Ra-values in both the bent and straight wires (*p* < 0.000) (Fig. 4).

**Table 1:** Surface roughness, Young’s modulus, and maximum force of bent/straight wires before and after immersion

Groups		Surface roughness ×10^−2^ (μm)			Mean flexural young’s modulus (GPa)	Mean Maximum force (N)
		Ra-value	Ra	Rz		
1 (pH 5.6)	Bent	24.1 ± 4.5	30.2 ± 5.7	117.3 ± 43.4	295 ± 46.98	32.5 ± 3.24
	Straight	11.1 ± 2.5			505 ± 38.34	27.9 ± 1.07
2 (pH 6.6)	Bent	18.3 ± 3.8	11.5 ± 1.5	81.2 ± 4.8	300 ± 46.72	31.4 ± 5.86
	Straight	9.4 ± 2.9			502 ± 52.48	27.6 ± 1.40
3 (pH 7.6)	Bent	20.5 ± 4.2	14.0 ± 0.9	78.8 ± 8.5	242 ± 35.07	29.2 ± 7.43
	Straight	10.1 ± 2.4			485 ± 92.66	27.2 ± 3.00
4 (Control)	Bent	17.9 ± 2.2	4.4 ± 0.6	28.4 ± 6.8	345 ± 85.90	32.5 ± 3.72
	Straight	8.2 ± 0.9			496 ± 18.62	28.4 ± 0.42

Mean roughness (Ra-value), mean flexural Young’s modulus, and mean maximum force of bent/straight wires as well as mean roughness (Ra and Rz) of straight wires before (control group 4) and after immersion in artificial saliva at pH 5.6, 6.6, and 7.6 (experiment groups 1, 2, and 3). Data are shown as mean ± SD

Compared to the wires in control group, the surface roughness of the bent and straight wires immersed in pH 5.6 and 7.6 were significantly rougher (*p* < 0.000) (Table 1). The surface roughness of both wires at pH 5.6 was also significantly different from that of the wires at pH 6.6 and 7.6 (*p* < 0.05) (Table 1). There is no difference in surface roughness of wires between the control and pH 6.6 groups (*p* = 0.740). A bigger distribution of data for the immersion groups than for the control group was observed, where higher SD were noted for immersion groups than for control groups. Similarly, higher SD values were noted on bent wires than on straight wires (Table 1).

### Flexural young’s modulus

The flexural Young’s modulus of SS wires had been distinctively reduced after bending (*p* < 0.000). In the control group, Young’s modulus was 496 ± 18.62 GPa measured in the straight wires and 345 ± 85.90 GPa in the bent wires (Table 1). This discrepancy between the bent and straight wires was not diminished by immersion in artificial saliva of various pH. On the contrary, the discrepancy was enlarged as pH increased, which was mainly contributed by the decrease of Young’s moduli of bent wires because Young’s moduli of straight wires were hardly influenced by pH (*p* = 0.772) (Fig. 4). The lowest Young’s modulus measured in this study was 242 ± 35.07 GPa, marked by the bent wires immersed in pH 7.6 (Table 1).

### Maximum force values

The maximum force of the bent wires was significantly higher than that of the straight wires throughout the experiment groups and control group (*p* < 0.00). After wire bending, the maximum force increased from 28.4 ± 0.42 N to 32.5 ± 3.224 N (*p* < 0.05). After immersion in artificial saliva for 4 weeks, there was a mild decrease in maximum force of the straight wires as pH increased, whereas a dramatic drop in maximum force in bent wires was noticed (Fig. 4). Yet pH did not make significant influence on SS wires regarding the maximum force values in either the bent or straight wires (*p* = 0.127).

### Scanning electron microscopy

**Fig. 5:**
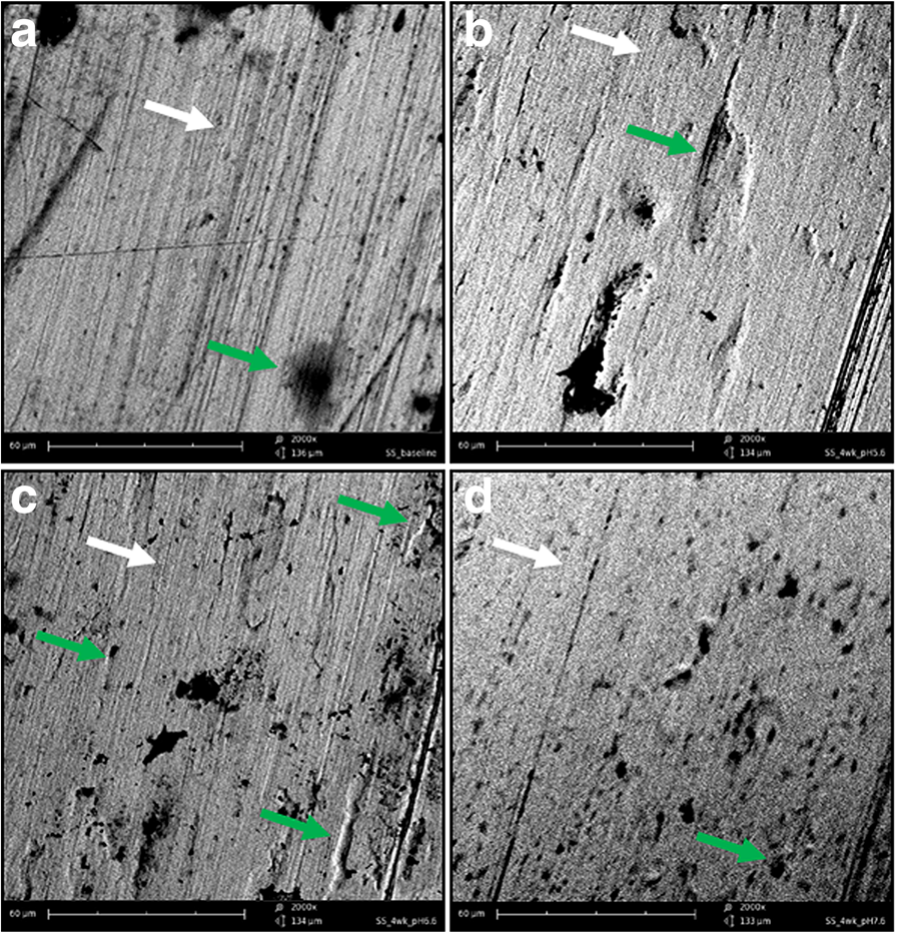
SEM images of straight rectangular SS wires at ×2000 magnification (**a–d**). Representative SEM images of SS wire of control group, pH 5.6, 6.6, and 7.6, respectively. *White arrows* indicate typical manufacturing striations; *green arrows* indicate specific surface irregularities

Scratches along the axial of wires were observed in all SEM images, which are typical defects of SS surface indicated by white arrows. The SEM image of SS wire from the control group showed a relatively smooth surface except for the typical striations and flaws existing on the wires from the experimental groups as well (Fig. 5a). The surface roughness of the wire from the control group had a mean Ra of 4.4 × 10^−2^ μm and Rz of 28.4 × 10^−2^ μm, which were the lowest values compared to the rest (*p* < 0.05) (Table 1).

Figure 5b depicts the surface of SS wire immersed in artificial saliva at pH 5.6 for 4 weeks. An increased corrosion pattern was observed compared to other pH groups. Irregularities on the surface were broader and the roughness peaks were bigger (green arrow). Scratch lines are more pronounced on the sample wire from this pH group than in the other pH groups (white arrow). These observations were in line with the measurements made upon SEM images, mean Ra and Rz were 30.2 × 10^−2^ μm and 177.3 × 10^−2^ μm, respectively, where the parameters from pH 5.6 group were higher than those from other pH groups (*p* < 0.05) (Table 1).

Figure 5c and d show the surface of SS wire immersed in artificial saliva at pH 6.6 and 7.6. These surfaces revealed more irregularities than the wire surface from the control group and those irregularities were smaller than those observed in Fig. 5b. In Fig. 5d, pores (green arrow) are seen in higher frequency than in the other pH groups. But they were smaller in diameter and depth than the surface irregularities in Fig. 5b. This was in accordance with a mean Rz of 78.8 × 10^−2^ μm and Ra of 14.0 × 10^−2^ μm (Table 1).

Although Rz was more sensitive than Ra in revealing surface roughness, both parameters showed that SS wires had the most rough surface after bending and immersion at pH 5.6 (*p* < 0.05) (Table 1). The influence of pH on surface roughness quantified by 3D construction of SEM images was consistent with the surface roughness measured by profilometer, which was much more obvious than observation done via a profilometer (Fig. 5). Especially, the Ra derived from SEM images was comparable to the measurements done by profilometer except that it was (11.1 ± 2.5) × 10^−2^ μm as measured by profilometer and (30.2 ± 5.7) × 10^−2^ μm by 3D reconstruction of SEM images (Table 1).

## Discussion

### Surface properties

Determining the surface roughness of stainless steel wires is a fundamental concern in corrosion resistance of a wire for orthodontic clinic. Straight archwires are hardly used in a patient’s mouth, and wire bending is an integral part of the orthodontic treatment. Even though the preformed SS archwires are popularly used nowadays, the chairside wire bending is inevitable due to the variations in the malocclusions of individual patient. In this research it is determined that bent SS wires have a highly significantly rougher surface than straight SS wires, ignoring pH. These divergences in surface roughness between bent and straight wires could be caused by the use of pliers when bending the wires, which could easily cause damages to the protective oxide layer.

In this research, Ra-values revealed that bent wires immersed in pH 5.6 and 7.6 solutions have a significantly rougher surface than bent wires at baseline. The increase of surface roughness by decreasing the pH is in agreement with results of other study. However, the surface of SS wires has also been roughened by artificial saliva at pH 7.6 according to our experiment. So far, no researchers had investigated the influence of basic saliva on SS archwires. This phenomenon of basic solution corroding SS wire was minute on straight wires and was amplified on bent wires, which has been echoed by images and measurements of SEM. Hence, it is recommendable that more research should be done regarding immersion in artificial saliva at basic pH because of its clinical relevance that the pH of resting saliva ranges from pH 5.6 to 7.6 [9, 10]. On the other hand, pH 6.6 seemed to be an optimal pH for SS archwire to perform and thus this might be our goal to balance oral pH especially during orthodontic treatment.

### Mechanical properties

Mechanical properties of SS wires are key to understand the wire application clinically since it determines the force generated for tooth movement. It has been reported that a flexural Young’s modulus of ~133–184 GPa for as-received SS archwires was measured by a three-point flexural test [13], while 468–526 GPa of Young’s modulus on SS wires was measured in this study by a four-point bending test, which was about threefold of the former. The explanation for the differences could be the support span. A support span of 20 mm was used in this research (Fig. 5), while other researchers used a lower support span of 14 and 9 mm [14, 20]. Alteration of the support span alters the load-deflection rate, even as alteration of the wire diameter [21]. Another explanation might be that smaller cross section increases the wire stiffness [2]. In other research, the wires were tested on their 0.64-mm side, whereas they were tested on their 0.43-mm side in this research [13].

In our study, we found that wire bending played an essential role on the mechanical properties of SS wires. It lowered Young’s modulus, and this influence was enhanced by the increase of pH. A higher flexural Young’s modulus indicates a stiffer wire with a greater resistance to deformation. Wire bending was originally introduced to elongate the length of a wire and thus to increase the flexibility and to lower Young’s modulus in order to deliver a moderate and persistent optimal force for tooth movement. As our finding pointed out that bending discounts the flexibility and Young’s modulus of the material, it might have been the effective elongation that guarantees a desired result on this bending of wires.

Regarding the influence of corrosion on mechanical properties of bent and straight SS archwires, it is revealed that bent wires became vulnerable to pH and the mechanical properties were altered.

## Conclusions

Bending has a significant influence on surface roughness and mechanical properties of rectangular SS archwires. pH plays a synergistic effect on the change of mechanical properties of SS wires along with wire bending.

The clinical implications of this study are as follows: (1) salivary pH tests for patient with fixed orthodontic appliance should be recommended and (2) bending on SS archwires should be done carefully for patients with imbalanced salivary pH (especially for basic pH) due to the synergistic effect.
